# Iwr1 Protein Is Important for Preinitiation Complex Formation by All Three Nuclear RNA Polymerases in *Saccharomyces cerevisiae*

**DOI:** 10.1371/journal.pone.0020829

**Published:** 2011-06-10

**Authors:** Anders Esberg, Zarmik Moqtaderi, Xiaochun Fan, Jian Lu, Kevin Struhl, Anders Byström

**Affiliations:** 1 Department of Molecular Biology, Umeå University, Umeå, Sweden; 2 Department of Biological Chemistry and Molecular Pharmacology, Harvard Medical School, Boston, Massachusetts, United States of America; Institute of Developmental Biology and Cancer Research, France

## Abstract

**Background:**

Iwr1, a protein conserved throughout eukaryotes, was originally identified by its physical interaction with RNA polymerase (Pol) II.

**Principal Findings:**

Here, we identify Iwr1 in a genetic screen designed to uncover proteins involved in Pol III transcription in *S. cerevisiae*. Iwr1 is important for Pol III transcription, because an *iwr1* mutant strain shows reduced association of TBP and Pol III at Pol III promoters, a decreased rate of Pol III transcription, and lower steady-state levels of Pol III transcripts. Interestingly, an *iwr1* mutant strain also displays reduced association of TBP to Pol I-transcribed genes and of both TBP and Pol II to Pol II-transcribed promoters. Despite this, rRNA and mRNA levels are virtually unaffected, suggesting a post-transcriptional mechanism compensating for the occupancy defect.

**Conclusions:**

Thus, Iwr1 plays an important role in preinitiation complex formation by all three nuclear RNA polymerases.

## Introduction

Eukaryotic cells contain three multi-subunit RNA polymerases (Pol) that transcribe the nuclear genome and are responsible for the production of selected classes of RNAs [1]–[5]. Pol I is responsible for synthesis of the tandem repeated ribosomal RNA genes, Pol II synthesizes mRNA and many non-coding RNAs, and Pol III synthesizes tRNA, 5S rRNA, and few other small untranslated RNAs. These RNA polymerases share 5 subunits, and their catalytic cores are similar to each other and to *E.coli* RNA polymerase [6]. Unlike bacterial and bacteriophage RNA polymerases that bind specifically to promoter sequences, the eukaryotic enzymes work in conjunction with transcription factors that directly bind promoters and recruit the appropriate RNA polymerase to initiate transcription [7]. The TATA-binding protein (TBP) is required for transcription by all three RNA polymerases [8], and it is a component of multi-protein complexes that function specifically with a particular RNA polymerase machinery [9].

Despite the similarities between RNA polymerases and the common requirement for TBP, the Pol II and Pol III transcription machineries are mechanistically distinct. Pol II core promoters consists of TATA, initiator, and downstream elements that are recognized by the basal transcription machinery that contains TBP, Pol II, and general transcription factors [10]. Upon initiation, Pol II dissociates from these general factors and associates with “elongation factors” that travel down the mRNA coding region [11]. *In vivo*, efficient transcription requires activator proteins that bind specifically to regulatory DNA sequences and, via co-activators, stimulate the basal transcription machinery [7]. Some Pol II-transcribed genes are regulated by repressors that bind to specific DNA sequences. The identity, quality, and location of regulatory sequences are gene-specific, with the consequence that every gene has a distinct pattern of expression.

For the vast majority of Pol III-transcribed genes, promoter recognition elements are located internally within the RNA coding region, and Pol III transcription involves a multi-step assembly of general initiation factors [1], [4], [12]. In general, the six-subunit TFIIIC binds to the A- and B-boxes, and it acts as an assembly factor directing binding of the TBP complex, TFIIIB, to a position just upstream of the initiation site. Transcription of 5S rRNA genes requires an additional factor, TFIIIA, that binds to the A-box, C-box, and IE-element. Once TFIIIB is assembled, the RNA polymerase is recruited and directs multiple rounds of transcription. Although Pol III genes are not individually regulated in the manner of Pol II genes, they are collectively subject to the negative regulator Maf1, which inhibits transcription in response to stress signals such as oxidative stress, cell wall stress, DNA damage, or nutrient limitation [13].

For the vast majority of Pol III-transcribed genes, promoter recognition elements are located internally within the RNA coding region, and Pol III transcription involves a multi-step assembly of general initiation factors [1], [4], [12]. In general, the six-subunit TFIIIC binds to the A- and B-boxes, and it acts as an assembly factor directing binding of the TBP complex, TFIIIB, to a position just upstream of the initiation site. Transcription of 5S rRNA genes requires an additional factor, TFIIIA, that binds to the A-box, C-box, and IE-element. Once TFIIIB is assembled, the RNA polymerase is recruited and directs multiple rounds of transcription. Although Pol III genes are not individually regulated in the manner of Pol II genes, they are collectively subject to the negative regulator Maf1, which inhibits transcription in response to stress signals such as oxidative stress, cell wall stress, DNA damage, or nutrient limitation [13].

After transcription, specific nucleosides in primary tRNA transcripts become modified to yield a mature functional tRNA [14]. In *S. cerevisiae*, the initiator methionine tRNA 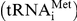 contains a unique 2′-O-ribosyl phosphate modification (Ar(p)) at position 64 [15] that is important for the discrimination between translational initiation and elongation [16]–[18]. Rit1, the initiator 2′-O-ribosyl phosphate transferase, is not required for normal cell growth, but a synergistic growth defect is observed in a *rit1* deletion strain also containing a reduced number of initiator methionine tRNA (*IMT*) genes [19].

Using a genetic screen based on synthetic lethality in a *rit1* mutant background to identify genes important for Pol III transcription, we have isolated mutations in the *IWR1* gene. This was unexpected, because Iwr1 was originally identified by its physical association with Pol II [20], [21], and it affects the basal and regulated expression of specific Pol II-transcribed genes [22], presumably through a direct effect on importing Pol II into the nucleus [23]. We show that Iwr1 is important for Pol III transcription, as an *iwr1* mutant strain shows reduced association of TBP and Pol III at Pol III promoters, a decreased rate of Pol III transcription, and lower steady-state levels of Pol III transcripts. In addition, we show that Iwr1 is important for association of TBP to the Pol I-transcribed rDNA locus and for recruitment of TBP and Pol II to Pol II-transcribed loci. These data suggest that Iwr1 plays an important role in preinitiation complex formation by all three nuclear RNA polymerases in yeast.

## Materials and Methods

### Screen for mutants that require the *RIT1* gene for growth

The genetic screen utilized to identify mutants requiring *RIT1* for growth was based on a colony sectoring assay as described previously [24]. Candidate synthetic-lethal strains were crossed to UMY2395 and investigated for dominance/recessiveness and for 2∶2 segregation of the non-sectoring phenotype. A YCp50 genomic library was used to transform *iwr1-2* (UMY2299), *rpc160-101* (UMY2304), and *rpb5-101* (UMY2309) mutants, and transformants that could lose the *RIT1* plasmid were identified. To confirm that the mutations in UMY2299 and UMY2304 were genetically linked to the *IWR1* and *RPC160* loci, we integrated a *URA3* marker at the corresponding wild-type locus in *rit1*Δ strains, generating UMY2448 and UMY2332. These strains were crossed to each mutant (UMY2299 and UMY2304) and tetrad analysis showed co-segregation of the Ura^+^ and sectoring phenotypes.

### Strains and DNAs

The source and genotypes of yeast strains used in this study are listed in table 1. Yeast strains and DNA molecules were constructed by standard methods, and the details are provided in Methods S1. Sequence analyses on chromosome IV revealed that *iwr1-1* has an insertion of an adenine at position 255085, the *iwr1-2* mutant allele carries an insertion of a thymine at position 254821, and in the *iwr1-3* mutant there is a substitution from a guanine to a thymine at position 254368.

**Table 1:** Yeast strains used in this study.

Strain	Genotype	Source
UMY2219	*MATa ura3-1 leu2-3, 112 trp1-1 ade2-1 ade3::hisG his3-11,15 can 1-100*	This lab.
UMY2220	*MATα ura3-1 leu2-3, 112 trp1-1 ade2-1 ade3::hisG his3-11,15 can 1-100*	This lab.
UMY2366	*MATa/MATα ura3-1/ura3-1 leu2-3, 112/leu2-3, 112 trp1-1/trp1-1 ade2-1/ade2-1 ade3::hisG/ade3::hisG his3-11,15/his3-11,15 can 1-100/can 1-100*	
UMY2395	*MATa ura3-1 leu2-3, 112 trp1-1 ade2-1 ade3::hisG his3-11,15 can 1-100 rit1::TRP1*	This study
UMY2396	*MATα ura3-1 leu2-3, 112 trp1-1 ade2-1 ade3::hisG his3-11,15 can 1-100 rit1::TRP1*	This study
UMY2418	*MATα ura3-1 leu2-3, 112 trp1-1 ade2-1 ade3::hisG his3-11, 15 can 1-100 rit1::TRP1* p1119	This study
UMY2316	*MATα ura3-1 leu2-3, 112 trp1-1 ade2-1 ade3::hisG his3-11, 15 can 1-100 rit1::TRP1 iwr1-1* p1119	This study
UMY2299	*MATα ura3-1 leu2-3, 112 trp1-1 ade2-1 ade3::hisG his3-11, 15 can 1-100 rit1::TRP1 iwr1-2* p1119	This study
UMY2312	*MATα ura3-1 leu2-3, 112 trp1-1 ade2-1 ade3::hisG his3-11, 15 can 1-100 rit1::TRP1 iwr1-3* p1119	This study
UMY2450	*MATα ura3-1 leu2-3, 112 trp1-1 ade2-1 ade3::hisG his3-11, 15 can 1-100 rit1::TRP1 iwr1-1* p1119	This study
UMY2451	*MATα ura3-1 leu2-3, 112 trp1-1 ade2-1 ade3::hisG his3-11, 15 can 1-100 rit1::TRP1 iwr1-2* p1119	This study
UMY2452	*MATα ura3-1 leu2-3, 112 trp1-1 ade2-1 ade3::hisG his3-11, 15 can 1-100 rit1::TRP1 iwr1-3* p1119	This study
UMY2808	*MATα ura3-1 leu2-3, 112 trp1-1 ade2-1 ade3::hisG his3-11, 15 can 1-100 iwr1-2*	This study
UMY2448	*MATa ura3-1 leu2-3, 112 trp1-1 ade2-1 ade3::hisG his3-11, 15 can 1-100 rit1::TRP1 IWR1::*pRS306-*IWR1*	This study
UMY2304	*MATα ura3-1 leu2-3, 112 trp1-1 ade2-1 ade3::hisG his3-11, 15 can 1-100 rit1::TRP1 rpc160-101* p1119	This study
UMY2469	*MATα ura3-1 leu2-3, 112 trp1-1 ade2-1 ade3::hisG his3-11, 15 can 1-100 rit1::TRP1 rpc160-101* p1119	This study
UMY2802	*MATα ura3-1 leu2-3, 112 trp1-1 ade2-1 ade3::hisG his3-11, 15 can 1-100 rpc160-101*	This study
UMY2332	*MATa ura3-1 leu2-3, 112 trp1-1 ade2-1 ade3::hisG his3-11, 15 can 1-100 rit1::TRP1 RPC160::pRS306-RPC160*	This study
UMY2309	*MATα ura3-1 leu2-3, 112 trp1-1 ade2-1 ade3::hisG his3-11, 15 can 1-100 rit1::TRP1 rpb5-101* p1119	This study
UMY2426	*MATα ura3-1 leu2-3, 112 trp1-1 ade2-1 ade3::hisG his3-11, 15 can 1-100 rit1::TRP1 rpb5-101* p1119	This study
UMY2804	*MATα ura3-1 leu2-3, 112 trp1-1 ade2-1 ade3::hisG his3-11, 15 can 1-100 rpb5-101*	This study
UMY2984	*MATα ura3-1 leu2-3, 112 trp1-1 ade2-1 ade3::hisG his3-11, 15 can 1-100 rpc160-101 iwr1-2* p1251	This study
UMY2986	*MATα ura3-1 leu2-3, 112 trp1-1 ade2-1 ade3::hisG his3-11, 15 can 1-100 rpb5-101, iwr1-2* p1251	This study
UMY2975	*MATα ura3-1 leu2-3, 112 trp1-1 ade2-1 ade3::hisG his3-11, 15 can 1-100 maf1*Δ	This study
UMY3059	*MATα ura3-1 leu2-3, 112 trp1-1 ade2-1 ade3::hisG his3-11, 15 can 1-100 maf1*Δ *iwr1-2*	This study
UMY3032	*MATα ura3-1 leu2-3, 112 trp1-1 ade2-1 ade3::hisG his3-11, 15 can 1-100 IWR1-3HA*	This study
UMY3034	*MATα ura3-1 leu2-3, 112 trp1-1 ade2-1 ade3::hisG his3-11, 15 can 1-100 IWR1-13MYC*	This study
UMY3031	*MATa ura3-1 leu2-3, 112 trp1-1 ade2-1 ade3::hisG his3-11, 15 can 1-100 RPC160-3HA*	This study
UMY3241	*MATα ura3-1 leu2-3, 112 trp1-1 ade2-1 ade3::hisG his3-11, 15 can 1-100 RPC160-3HA iwr1-2*	This study
UMY3035	*MATα ura3-1 leu2-3, 112 trp1-1 ade2-1 ade3::hisG his3-11, 15 can 1-100 IWR1-13MYC RPC160-3HA*	This study
BY4741	*Mat a his3*Δ*1 leu2*Δ*0 met15*Δ*0 ura3*Δ*0*	Invitrogen
RN3812	*Mat a his3*Δ *1 leu2*Δ*0 met15*Δ*0 ura3*Δ*0 iwr1::KanMX4*	Invitrogen

### Immunofluorescence

To localize Iwr1, cells were grown in 5 ml YEPD at 30° to an OD_600_ of 0.3, 670 µl formaldehyde (37%) was added and the cells were incubated for 40 min at RT. Cells were collected and washed once with 1× PBS, pH 7.4. The primary antibody, mouse anti-HA (12CA5), was diluted 1∶2000, and the secondary antibody, goat anti-mouse linked to Cy3 (PA43002, Amersham Biosciences), was diluted 1∶200. Cells were viewed in a Zeiss Axioskope 50 microscope using a 100× objective. Images were acquired using a Hamamatsu-digital camera (C4742-95).

### Polysome profiles

Cells were grown in 200 ml at 30° in selective medium to an OD_600_ 0.4. Cycloheximide was added (100 µg/ml) 5 min before transferring the culture to an ice water bath for 15 min. Cells were collected at 4°, washed twice in ice-cold Breaking buffer (Bb; 20 mM Tris-HCl pH 7.4, 10 mM MgCl_2_, 100 mM KCl, 0.5 mM DTT, 100 µg/ml cycloheximide). The cells were resuspended in 1 volume of Bb, followed by addition of 1 volume of glass beads, and cells were disrupted by 6×20 sec on a vortex mixer, and the insoluble material was pelleted by centrifugation at 10,000×g for 5 min at 4°. The supernatant was transferred to a microfuge tube and subjected to a second centrifugation at 10,000×g for 20 min at 4°. The supernatant was applied to a 12 ml linear 10 to 45% sucrose gradient prepared in Bb lacking cycloheximide and centrifuged for 2.5 hrs at 40,000 rpm in a SW41 rotor at 4°. The gradients were collected from the top, and A_254_ absorbances were monitored with the ISCO detection system.

### RNA analysis

Northern blots were performed by standard methods using the following oligonucleotides for detection: GGACATCAGGGTTATGAGCC- 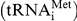; TGCTCCAGGGGAGGTTCGAAC 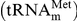;GCGTTGTTCATCGAT (5.8S rRNA), and the levels of RNA quantified by phosphorimager analysis. For direct measurements, gels were stained with ethidium bromide, and RNA were quantified by using a BioRad Fluor-S™ MultiImager and the QuantityOne-4.2.3 software. To analyze RNA levels by quantitative reverse-transcriptase PCR, total RNA was treated with DNase I on Qiagen RNeasy columns, and first-strand cDNA synthesis was performed with random hexamers and Superscript III reverse transcriptase (Invitrogen) on 1 µg of total RNA. The relative representation of specific loci in this material was assayed by quantitative PCR in real-time on an Applied Biosystems 7500 machine.

### *In vivo* labeling of RNA

In labeling experiments, cells were grown to an OD_600_ of 0.8 in 120 ml SC-uracil medium at 30° before 125 µCi of ^3^H-Uridine was added (33 Ci/mmol, Amersham Biosciences). Samples (20 ml) were collected after 0, 5, 10, 20, and 40 min. Total RNA was prepared and separated on an 8% polyacrylamide 8 M urea gel. The gel was stained with ethidium bromide, quantified, soaked in NAMP100 Amplifier for 15 min (Amersham Biosciences), dried onto 3 MM Whatman paper, and exposed to film. Signals were quantified using QuantityOne-4.2.3 software (BioRad), and the rate of synthesis for each RNA type was calculated. For the pulse-chase experiment, cells were grown to an OD_600_ of 0.8 in 50 ml SC-uracil medium at 30° and pulse-labeled using 125 µCi of ^3^H-Uridine (33 Ci/mmol Amersham Biosciences) for 45 min. Cells were collected and resuspended in 250 ml pre-warmed SC medium containing excess (2 mM) uracil to begin the chase. Samples (40 ml) were collected after 0, 1, 2, 3, and 4 hrs. RNA was prepared, separated, and quantified as described above.

### Immunoprecipitation

50 µg total protein in buffer 1 (0.15 M Tris-HCl, pH 7.8, 50 mM KAc, 20% glycerol, 1 mM EDTA, 1× Protease inhibitors, 0.5 mM DTT) was incubated for 2 hrs with agarose beads (Sepharose 4 Fast Flow, Amersham Biosciences) in a rotating chamber. Beads were recovered and the supernatant were transferred to tubes containing agarose beads linked to either anti-HA or anti-MYC antibody (Sigma A2095 or M5546), and incubated 2 hrs at 4° in a rotating chamber. Beads were recovered and washed six times using 1 ml of buffer 2 (0.15 M Tris-HCl, pH 7.8, 50 mM KAc, 20% glycerol, 1 mM EDTA, 1× Protease inhibitors, 0.5 mM DTT, 0.5% Triton-X100), and proteins bound to the beads were recovered by incubating samples at 100° for 3 min in 1× loading buffer. Recovered tagged proteins were detected using standard western blot techniques.

### Chromatin immunoprecipitation

Yeast strains BY4741 and the isogenic strain containing an *iwr1* null allele RN3812 were obtained from Invitrogen. Cells were crosslinked with 1% formaldehyde and total chromatin was sonicated to an average size between 300–500 bp as described previously [25]. Chromatin immunoprecipitation was performed using antibodies against Tfc4, Rpc34 (both antibodies kindly supplied by Steve Hahn), Bdp1 (kindly provided by Ian Willis), and Rpb1 (8WG16 antibody from Covance). Immunoprecipitated DNA and total input control DNA were assayed by real-time quantitative PCR using the Applied Biosystems 7500 Real-time PCR System. Immunoprecipitation efficiency was determined for each locus by dividing the yield of PCR product in the immunoprecipitation sample by the amount of product obtained from the input control. Relative occupancy values were determined by dividing the immunoprecipitation efficiency at each locus by the immunoprecipitation efficiency at a negative control locus (either the middle of the *POL1* ORF or an ORF-free region of chromosome V). The occupancy value of the negative control, 1.0, was subtracted from all values to yield a baseline of 0. All occupancy values were normalized to set the occupancy of each factor in the wild-type strain at *tC(GCA)B* locus equal to 100 units. All experiments were performed a minimum of three times.

## Results

### Strains with a mutation in *IWR1* require the *RIT1* gene for growth

We reasoned that screening for mutations that are synthetic-lethal in combination with a *rit1*Δ mutant might identify genes required for efficient Pol III transcription. A *rit1*Δ mutant is viable with no apparent growth defects unless the steady-state level of 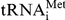 is reduced by deleting *IMT* genes, whereupon severe/modest growth defects are observed [19]. The genetic screen that was employed here to identify mutants requiring Rit1 for growth has been described in detail [24], [26]. Briefly, a *rit1* null strain carrying a plasmid with the *RIT1* gene was mutagenized, and colonies were screened for the inability to lose the *RIT1* plasmid. Thirty-two strains were identified that required the *RIT1* gene for survival. Here we describe one complementation group consisting of three slow growing and temperature sensitive (ts) mutants.

**Figure 1:**
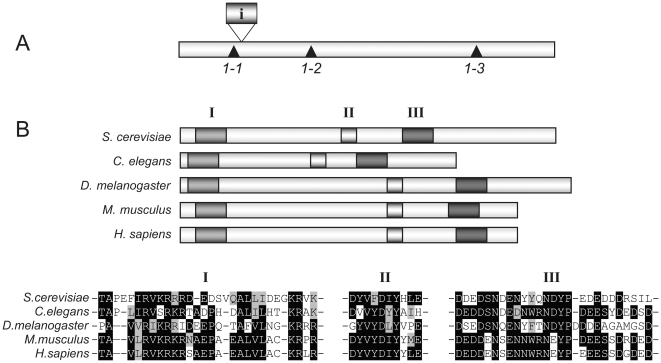
Characterization of the *IWR1* gene. (A) Positions of the *iwr1* mutations, *1-1*, *1-2*, and *1-3* are indicated with ▴, along with the position of the intron (i). (B) Putative Iwr1 homologues with the following accession numbers: *Saccharomyces cerevisiae IWR1* (*NP_010168*); *Caenorhabditis elegans*, *CAB03456*; *Drosophila melanogaster*, *NP_524940.1*; *Mus musculus*, *XP_134478*; *Homo sapiens*, *NP_115554*. Three regions of high similarity are indicated with I, II, and III. (C) Sequence comparison of regions I, II, and III in the indicated species.

To identify the gene mutated, a genomic library was introduced into one of the mutants, and plasmids complementing the *RIT1* dependence, ts, and slow growth phenotypes were identified. DNA sequencing and sub-cloning showed that the *IWR1* gene was responsible for the complementation. Genetic linkage between *IWR1* and the three original *iwr1* mutations was confirmed by targeted integration and tetrad analysis. To identify and characterize the *iwr1* mutations, we cloned and sequenced the gene from the three different mutant alleles. All three *iwr1* mutations generated truncated Iwr1 proteins of various lengths (Fig. 1A) (see Materials and Methods). When yeast Iwr1 was compared to translated ORFs from multi-cellular eukaryotes, potential homologues were identified. All identified proteins shared three distinct regions with high similarity (Fig. 1B). Thus, the *IWR1* gene encodes a conserved protein that is required for viability in a *rit1* null strain.

### The *iwr1-2* mutant has reduced Pol III transcripts and a defect in initiation of translation

**Figure 2:**
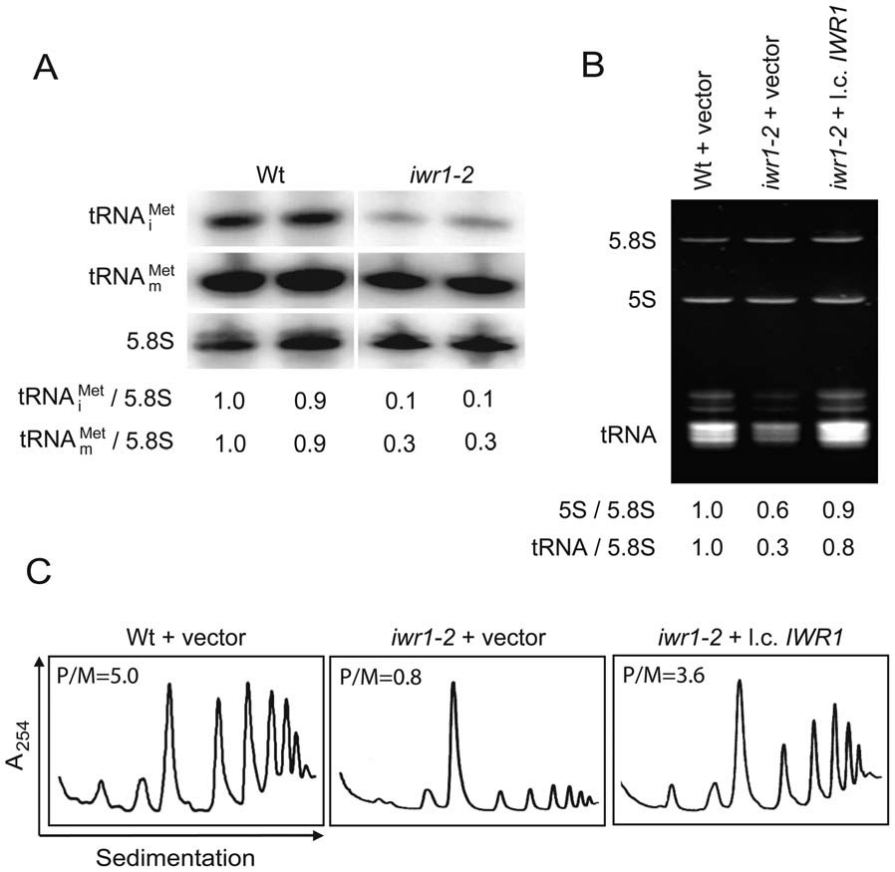
The *iwr1-2* mutant is defective for Pol III transcription and translational initiation. (A) Northern blots of RNA isolated from wild-type (UMY2220) and *iwr1-2* (UMY2808) strains that were probed for 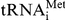, 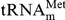, and 5.8S rRNA. (B) Ethidium bromide staining of total RNA from wild-type (UMY2220) carrying pRS316 and *iwr1-2* (UMY2808) strains carrying pRS316 or pRS316-*IWR1*. Band intensities were normalized to the 5.8S rRNA signals, and the amount of each RNA species was expressed relative to the corresponding value in the wild-type strain, which was set to 1. (C) Polysome profiles of total extracts isolated from wild-type (UMY2220) carrying pRS316, and *iwr1-2* (UMY2808), carrying pRS316 or pRS316-*IWR1* were analyzed by sedimentation in sucrose gradients. The polysome to monosome ratio (P/M) was calculated.

A *rit1* null strain with two of the four *IMT* genes deleted displays a synergistic growth defect that is overcome by increased gene dosage of *IMT* [19]. Introduction of a high-copy number plasmid carrying *IMT4* into an *iwr1* mutant suppressed the *RIT1* dependence for growth (data not shown). Suppression was specific for *IMT* genes, as *iwr1* mutants with increased dosage of the *EMT5* gene, encoding elongator methionine tRNA 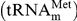, required *RIT1* for growth (data not shown). This suggests that the *RIT1* dependence of the *iwr1* mutant might be caused by reduced levels of 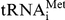. To investigate the level of 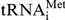 in the *iwr1-2* mutant, northern blot were conducted using total RNA isolated from wild-type and *iwr1-2* strains. The blots were probed for 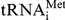, 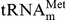, and the Pol I transcript 5.8S rRNA. The *iwr1-2* mutant showed a ten-fold reduction in the level of 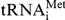 and a three-fold reduction of 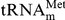 as compared with the isogenic wild-type (Fig. 2A).

As the levels of both 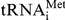 and 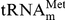 decreased in the *iwr1-2* mutant, we considered that all Pol III transcripts could be affected. Total RNA isolated from wild-type and *iwr1-2* strains was separated on a denaturing polyacrylamide gel, stained with ethidium bromide, and the total tRNA and 5S rRNA signals were quantified after normalization to 5.8S rRNA. The *iwr1-2* mutant showed a three-fold reduction in total tRNA levels and a 40% reduction in 5S rRNA compared to wild-type (Fig. 2B). We also investigated the levels of total tRNA and 5S rRNA in the *iwr1-1* and *iwr1-3* mutants and found that total tRNA was 64% and 22% reduced, and 5S rRNA was 35% and 31% reduced, respectively (data not shown). As reduced levels of 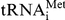 affect initiation of translation, we expected that the *iwr1-2* mutant should have a reduction in initiation of translation. Consistent with this idea, the *iwr1-2* mutant showed a six-fold reduction of polysome to monosome ratio when compared to wild-type (Fig. 2C). We conclude that the *iwr1* mutant has decreased levels of Pol III-transcribed RNAs, and it is the low level of 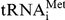 that likely causes the *RIT1* dependence and defect in translation initiation.

### Iwr1 is important for Pol III transcription

**Figure 3:**
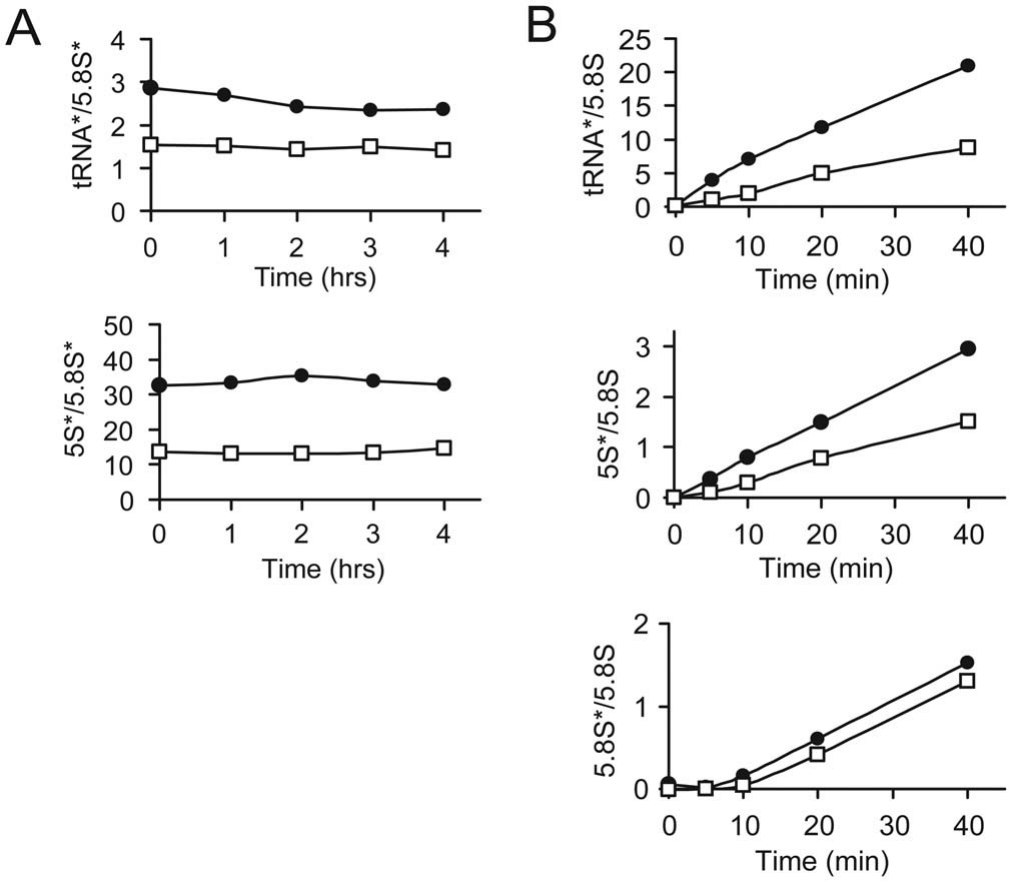
Iwr1 is required for efficient RNA polymerase III transcription. (A) To investigate the stability of tRNA (upper) and 5S rRNA (lower), total ^3^H-uridine labeled RNA were isolated from wild-type (UMY2220) and *iwr1-2* (UMY2808) strains carrying pRS316, labeled for 45 min with ^3^H-uridine and chased with excess of uracil for the indicated amount of time. The stability of tRNA and 5S rRNA in wild-type (•) and *iwr1-2* (□) strains is expressed relative to 5.8S rRNA. (B) To investigate rate of synthesis of Pol III transcription, Wild-type (UMY2220) and *iwr1-2* (UMY2808) strains carrying pRS316 were grown in SC-uracil media and labeled with ^3^H-uridine for the indicated time. The intensities of the ^3^H-signals were quantified as in (A) and normalized to the 5.8S rRNA ethidium bromide signal in the corresponding lane. The rate of synthesis for each RNA was calculated for wild-type (•), and *iwr1-2* mutant (□) strains (*-corresponds to ^3^H-labeled RNA signals). All experiments were performed two times.

The reduction in tRNA and 5S rRNA levels in the *iwr1* mutants could be caused by defects in stability, transcription, or a combination thereof. To test whether Iwr1 is needed for stability of Pol III-transcribed RNAs, a pulse-chase experiment was performed in wild-type and *iwr1-2* strains using ^3^H-uridine. Samples were taken at various times after the chase began, and total RNA was separated on a denaturing polyacrylamide gel. ^3^H-labeled tRNA and 5S rRNA were quantified after being normalized to the 5.8S rRNA. The *iwr1-2* mutant showed reduced levels of tRNA and 5S rRNA at time point 0; however, there was no further reduction during the 4 hr chase, indicating no decreased stability of tRNA and 5S rRNA (Fig. 3A). To investigate whether the *iwr1-2* mutation influences the rate of transcription by Pol III, wild-type and *iwr1-2* strains were cultured with ^3^H-uridine and samples removed at various time points after ^3^H-uridine addition and analyzed for tRNA, 5S rRNA, and 5.8S rRNA accumulation. In the *iwr1-2* mutant the incorporation of ^3^H-uridine into tRNA and 5S rRNA was reduced to 43% and 64% (*k*-value) (Fig. 3B) compared to wild-type, whereas the rate of 5.8S rRNA synthesis was virtually unaffected (94%). We conclude that Iwr1 is required for the efficient production of tRNA and 5S rRNA.

### The *IWR1* gene is genetically linked to the Pol III machinery

**Figure 4:**
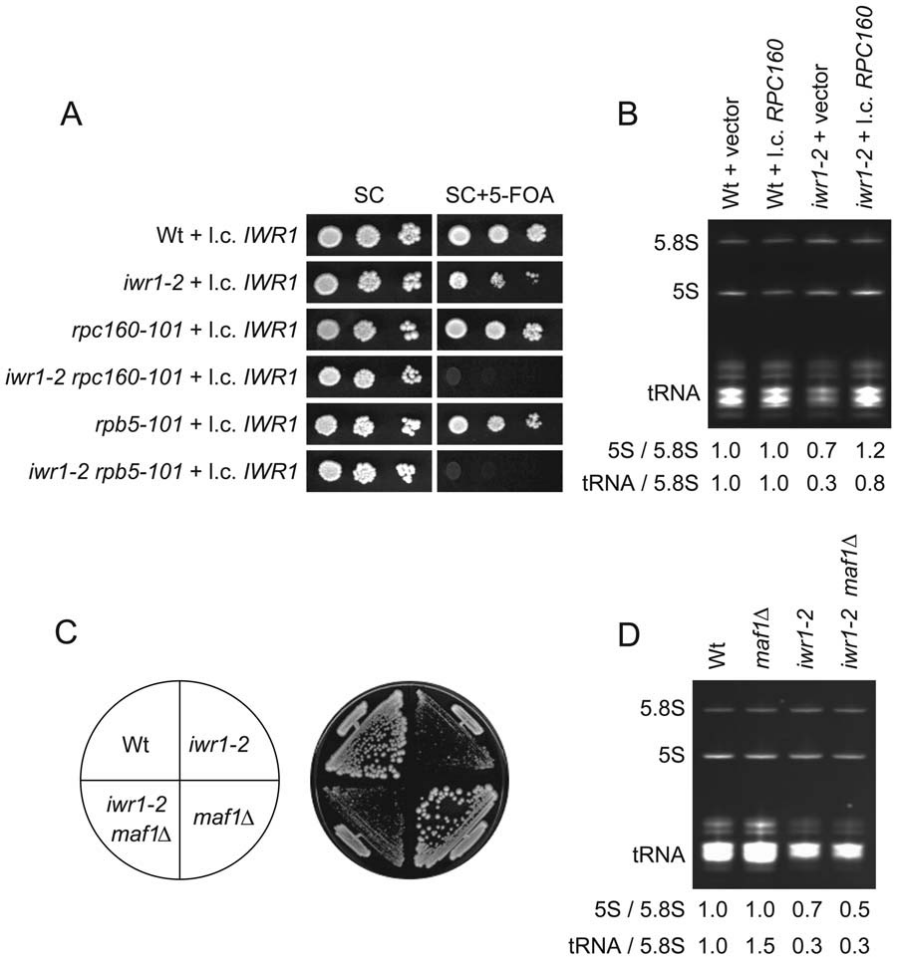
The *IWR1* gene is genetically linked to Pol III transcription. (A) Wild-type (UMY2220), *iwr1-2* (UMY2808), *rpc160-101* (UMY2802), *iwr1-2 rpc160-101* (UMY2984), *rpb5-101* (UMY2804), and *iwr1-2 rpb5-101* (UMY2986) strains all carrying pRS316-*IWR1* were grown in synthetic complete (SC) medium, serially diluted, spotted onto SC and SC+5-FOA plates. (B) Total RNA isolated from wild-type (UMY2220) and *iwr1-2* (UMY2808) strains carrying either pRS316 or pRS316-*RPC160* was analyzed as in Figure 2B. (C) Growth of wild-type (UMY2220), *maf1*Δ (UMY2975), *iwr1-2* (UMY2808), and *maf1*Δ *iwr1-2* (UMY3059) strains on YEPD plates. (D) Total RNA isolated from wild-type (UMY2220), *maf1*Δ (UMY2975), *iwr1-2* (UMY2808), and *maf1*Δ *iwr1-2* (UMY3059) strains was analyzed as in Figure 2B.

From the screen for mutants requiring the *RIT1* gene, we also obtained strains with mutations in the *RPC160* or the *RPB5* gene. The *RPC160* gene encodes the large subunit of RNA polymerase III, and the *RPB5* gene encodes one of five subunits that are shared among all three RNA polymerases [27]. In addition to the *RIT1* dependence, the *rpc160-101* and *rpb5-101* mutants showed a reduced growth rate and a 35% and 31% reduction in total tRNA levels, respectively (data not shown). To further investigate the role of Iwr1 in transcription by Pol III, we combined the *iwr1-2* allele with an *rpc160-101* or an *rpb5-101* mutation. The combinations were lethal, consistent with a role of Iwr1 in transcription by Pol III (Fig. 4A). Increased gene dosage of *RPC160*, but not *RPB5*, in the *iwr1-2* mutant suppressed the *RIT1* dependence and the low-level of total tRNA and 5S rRNA, and it partially suppressed the growth defect at 30° and 37° (data not shown, Fig. 4B).

Maf1 is the main regulator for signaling pathways to mediate repression of transcription by Pol III (For review see [13]. A *maf1* mutant is unable to repress transcription by Pol III in response to various stress signals. To investigate whether the reduced rate of Pol III transcription observed in the *iwr1-2* mutant is mediated through Maf1, we introduced a *maf1* null allele into the *iwr1-2* mutant. The double mutant showed similar growth phenotype (Fig. 4C) and steady-state levels of tRNA and 5S rRNA as the single *iwr1-2* mutant (Fig. 4D). Together these genetic data support a role for Iwr1 in transcription by Pol III and show that the *iwr1* mutant affects transcription by Pol III independently of the key negative regulator, Maf1.

### Iwr1 is a nuclear protein that may interact weakly with Pol III

**Figure 5:**
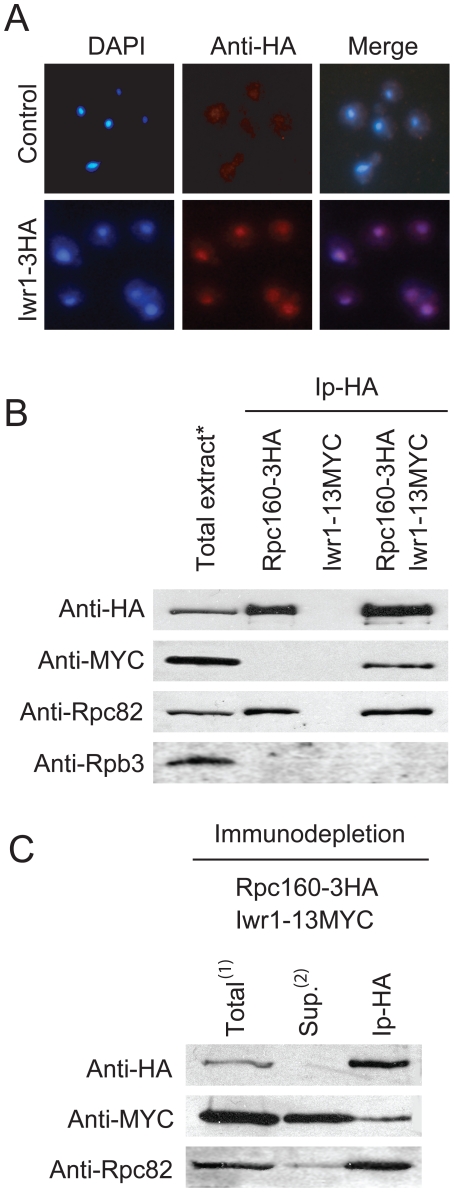
Iwr1 is predominantly a nuclear protein that may weakly interact with Pol III. (A) Localization of Iwr1-3HA using mouse anti-HA as primary antibody and goat anti-mouse linked to Cy3 as secondary antibody. Cells were stained with DAPI and viewed microscopically. (B) Immunoprecipitation analysis on protein extracts from *RPC160-3HA* (UMY3031), *IWR1-13MYC* (UMY3034), and *RPC160-3HA IWR1-13MYC* (UMY3035), (*-corresponds to total extract from the *RPC160-3HA IWR1-13MYC* strain). (C) Immunodepletion experiment of total protein extract from the *RPC160-3HA IWR1-13MYC* (UMY3035) strain (^1^-corresponds to 1/10 of input and ^2^-corresponds to 1/3 of supernatant.

To investigate the intracellular localization of Iwr1, we epitope-tagged the genomic copy of *IWR1* with the hemaglutanin epitope (HA). By indirect immunoflourescence, Iwr1-HA localized predominantly to the nucleus (Fig. 5A). Immunoprecipitation of HA-tagged Rpc160 co-precipitated Iwr1, suggesting a physical interaction between Iwr1 and Pol III (Fig. 5B). Gavin et al. (2002) have previously shown that Iwr1 co-purified with the Pol II specific subunit, Rpb3. However, no Rpb3 was detected in our Rpc160 immunoprecipitate, excluding the possibility that contaminating Pol II subunits were responsible for the presence of Iwr1 (Fig. 5B). Immunoprecipitation of a 13-myc-tagged Iwr1 co-precipitated Rpc160, but with very low efficiency (data not shown). By an Rpc160 immunodepletion experiment we showed that only 5–10% of total Iwr1 was associated with Rpc160 (Fig. 5C). To confirm that the Pol III complex remained intact throughout the experiment, we used the Pol III specific subunit Rpc82 as a control (Fig. 5C). Even though Iwr1 and Pol III do co-precipitate, the low level of association suggests an indirect or weak interaction.

### Reduced Pol III occupancy on tRNA genes in an *iwr1* mutant

**Figure 6:**
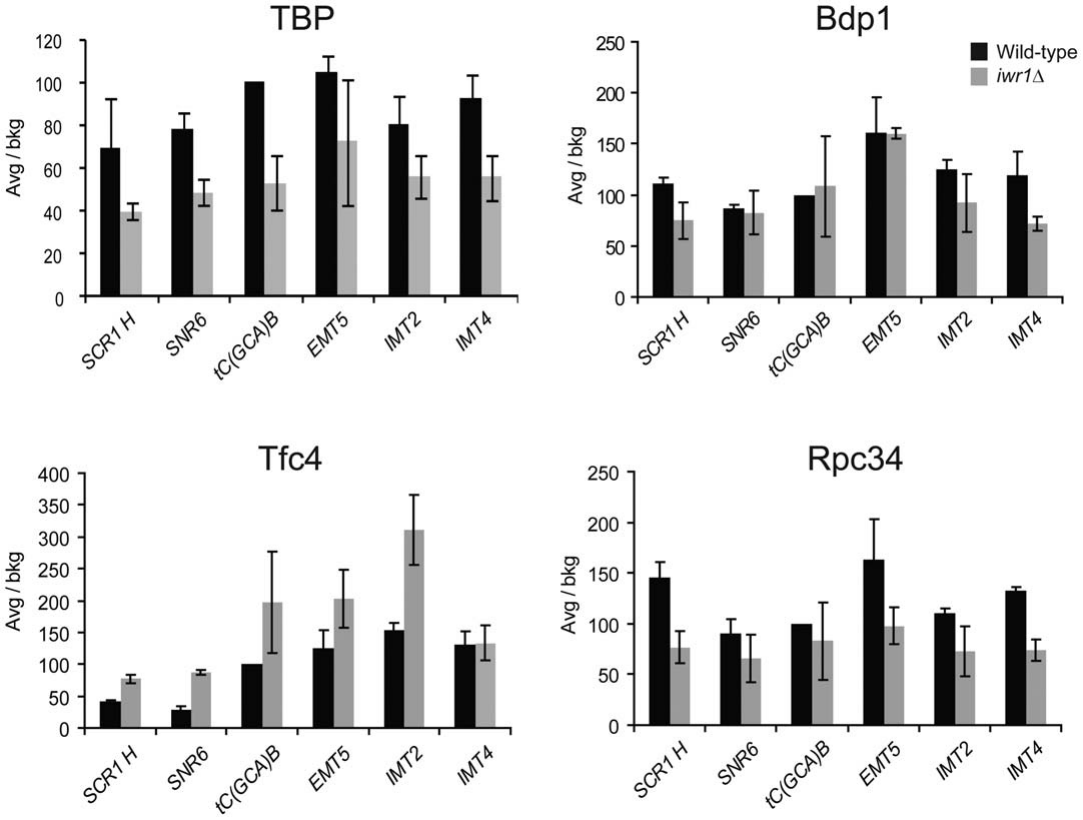
Occupancy profile of Pol III factors in the *iwr1* null strain. Chromatin immunoprecipitation was performed on chromatin from wild-type and *iwr1*Δ strains using antibodies against TBP, Bdp1, Tfc4, and Rpc34. Immunoprecipitation efficiency was determined for each Pol III locus by dividing the yield of PCR product in the immunoprecipitated sample by the amount of product obtained from the input control. Relative occupancy values were determined by dividing the immunoprecipitation efficiency at each locus by the immunoprecipitation efficiency at a negative control locus.

In addition to the polymerase, the Pol III transcription machinery consists of the multi-subunit transcription factors TFIIIB and TFIIIC [1], [4], [12]. TFIIIC consists of six subunits, which recognize the A- and B-boxes and promote recruitment of the initiation factor, TFIIIB, to the region upstream of the transcriptional start site. The recruited TFIIIB cooperates with TFIIIC to recruit the RNA polymerase to the transcriptional start site [28]. To investigate whether lack of Iwr1 function affects the Pol III transcription factor occupancy profile at Pol III-transcribed loci, we performed chromatin immunoprecipitation (ChIP) on wild-type and *iwr1Δ* strains using antibodies against the TFIIIB components TBP and Bdp1, the TFIIIC component Tfc4, and the Pol III-specific polymerase subunit Rpc34. We assayed the occupancy of each factor at a variety of Pol III loci: *SCR1H*, *SNR6*, *tC(GCA)B*, *EMT5*, *IMT2*, and *IMT4*. In the *iwr1*Δ strain, Bdp1 occupancy is essentially unchanged, TBP occupancy is reduced by 30 to 50%, Rpc34 occupancy is slightly decreased, and Tfc4 occupancy is normal to slightly increased (Fig. 6). This suggests that Iwr1 facilitates the recruitment of TBP and the RNA polymerase to Pol III-transcribed loci. We note that Bdp1 and TBP, which are both subunits of TFIIIB, show different occupancy profiles in the *iwr1*Δ strain; it is possible that deletion of Iwr1 affects the stability of the TBP-DNA association, while Bdp1 occupancy is maintained through its contacts with other components of the Pol III apparatus.

### Iwr1 is important for recruitment of TBP to Pol I, Pol II, and Pol III promoters

**Figure 7:**
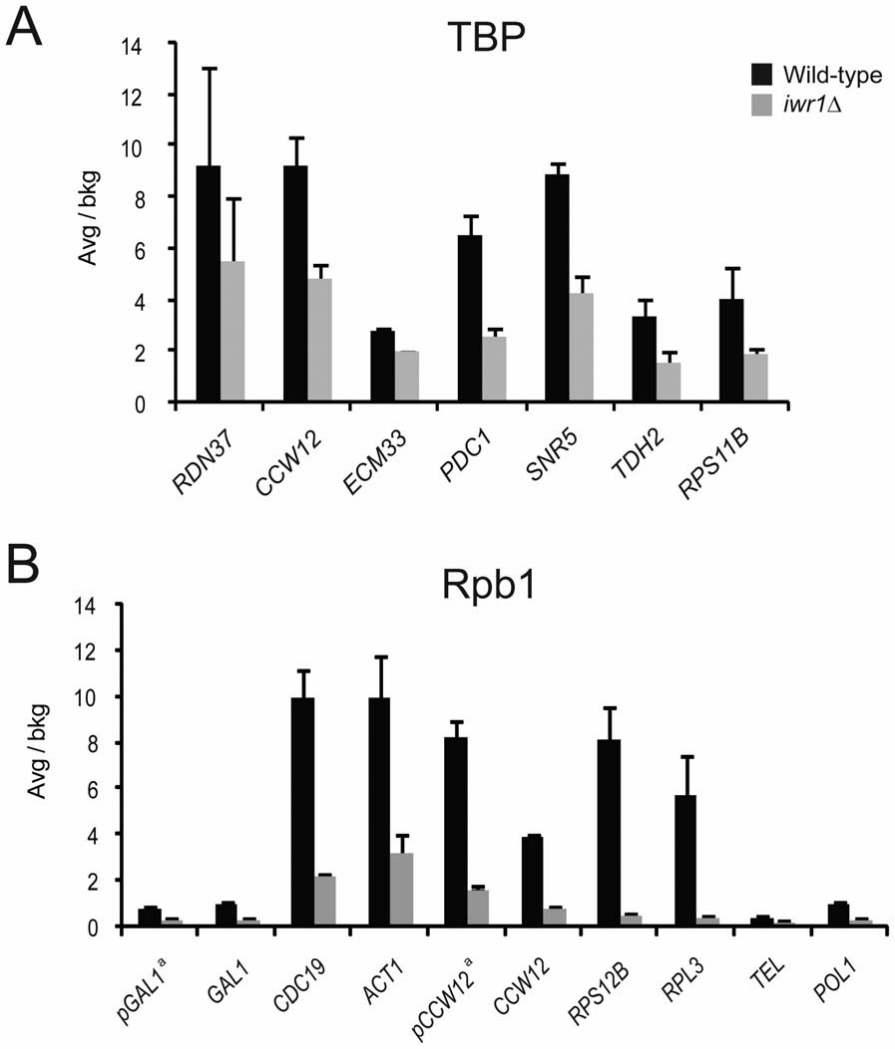
Occupancy profile of TBP and RNA polymerase in the wild-type and *iwr1* null strain. (A) TBP occupancy at the Pol I-transcribed RDN37 locus and at various Pol II-transcribed loci in wild-type and *iwr1Δ* strains. (B) Occupancy by the large subunit of RNA Polymerase II at various loci in wild-type and *iwr1Δ* strains. The occupancy values are expressed as folds over the values at an ORF-free control region. ^a^- corresponds to promoter region.

Because TBP participates in transcription by all three RNA polymerases and shows diminished occupancy at Pol III loci in the *iwr1Δ* strain, we also tested TBP occupancy by ChIP at Pol I- and Pol II-transcribed loci in wild-type and *iwr1Δ* strains. We found that TBP occupancy at Pol II loci is approximately two-fold lower in an *iwr1Δ* mutant strain, indicating that the *iwr1Δ* defect is not specific to Pol III transcription (Fig. 7A). Furthermore, occupancy by TBP at the Pol I-transcribed rDNA locus (*RDN37*) is also somewhat lower in the mutant strain (Fig. 7A). Iwr1 is therefore important for recruitment of TBP to loci transcribed by all three nuclear RNA polymerases in yeast.

### Pol II occupancy is reduced in the *iwr1Δ* mutant

In light of the known interaction between Iwr1 and RNA Pol II [20], [21] and the effect of the *iwr1Δ* mutation on TBP occupancy at Pol II-transcribed loci, we tested occupancy by the Pol II subunit Rpb1 in wild-type and *iwr1Δ* strains. We performed ChIP analysis on ten different Pol II-transcribed loci to determine the occupancy profile of Pol II in strains deleted for the *IWR1* gene. We observed a similar occupancy decrease on the Pol II loci as observed on Pol III-transcribed loci, i.e., recruitment of the polymerase was reduced in the *iwr1* null strain (Fig. 7B). Thus, two independent observations, namely reduced association of the polymerase and of TBP at Pol II-transcribed loci, strongly suggest that Iwr1 functions in transcriptional initiation by Pol II. After completion of our work, it was reported that Iwr1 is directly involved in the import of Pol II into the nucleus [23].

### The Pol III transcription defect is not caused by a Pol II transcriptional defect

**Figure 8:**
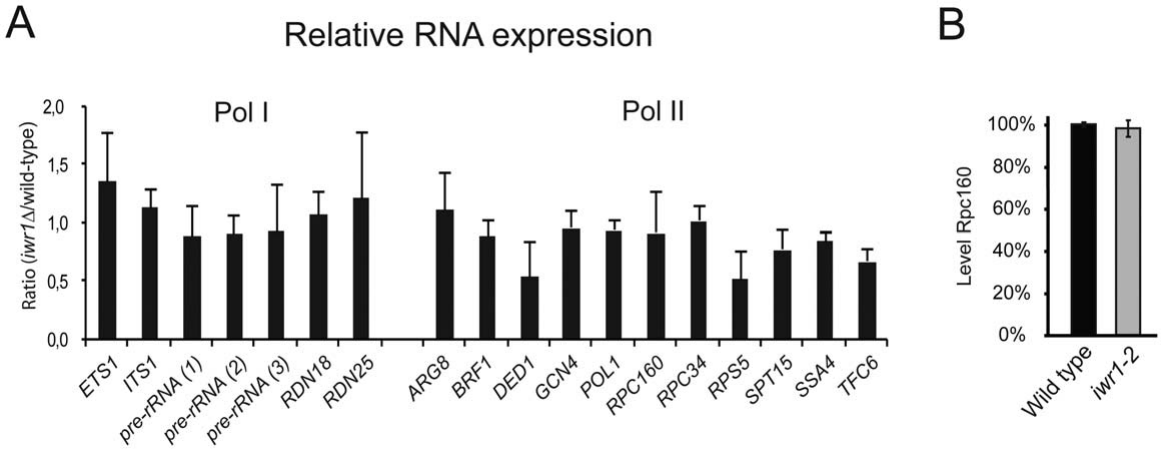
Relative expression of RNAs in *iwr1* and wild-type strains. (A) Relative expression of various regions of the long Pol I-transcribed region (left) and of a variety of Pol II-transcribed RNAs (right). The regions on the left were chosen to represent both precursor RNA (regions removed during RNA maturation) and mature RNA. (B) Western blot analysis of the *RPC160-3HA* (UMY3031) and the *iwr1-2 RPC160-3HA* (UMY3241).

To determine whether the polymerase occupancy decrease at Pol II-transcribed loci resulted in lower transcript levels of Pol II-transcribed genes, we analyzed the levels of eleven mRNAs by rtPCR (Fig. 8A). We included genes encoding components of the Pol III transcription machinery to determine whether the decreased abundance of these Pol II-transcribed factors might be the indirect cause of the observed defect in Pol III transcription. Strikingly, we did not observe significant differences in the steady-state level of RNA synthesized by Pol II in *iwr1* and wild-type cells (Fig. 8A). The TBP and polymerase recruitment defect at Pol II-transcribed loci in the *iwr1Δ* strain does not alter the steady-state level of the mRNAs tested, suggesting that a post-transcriptional mechanism is able to compensate for any resulting decrease in transcription. Furthermore, the normal levels of the mRNAs encoding the Pol III transcription factors Brf1, Tfc6, Rpc160, and Rpc34 make it clear that the observed decrease in transcription by Pol III in the *iwr1Δ* strain is not an indirect effect of diminished transcription of the Pol III machinery itself. In addition, western blot analysis of HA-tagged Rpc160 revealed no difference in the levels of Rpc160 between wild-type and *iwr1-2* strains (Fig. 8B). Thus, the defect in Pol III transcription in the *iwr1* mutant strain is not merely a trivial consequence of diminished Pol III transcription factor availability.

## Discussion

We isolated the *IWR1* gene in a genetic screen for mutants requiring the *RIT1* gene for growth. The *RIT1* gene encodes an initiator tRNA specific 2′-O-ribosyl phosphate (Ar(p)) modification enzyme [29], and the presence of the Ar(p) modification prevents 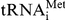 from being utilized in translation elongation [16]–[18]. Under conditions in which the 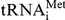 steady-state level is reduced, the Ar(p) modification is required to maintain a sufficient level of eIF2:GTP: 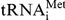 ternary complex to promote efficient translational initiation [19]. In the *iwr1-2* mutant the steady state level of 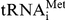 is reduced ten-fold, thereby dramatically affecting initiation of translation (Fig. 2A and C). In an *iwr1* mutant the *RIT1* gene is essential, as use of 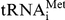 lacking Ar(p) for translation elongation would reduce the already limited pool of 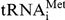 available for initiation to below the minimum level required for cell survival.

We found an *iwr1* mutant strain to have a significant reduction in steady state levels of tRNA and 5S rRNA. Interestingly, the large decrease in Pol III transcript levels in the *iwr1* strain (Fig. 2) occurs even though there is only a moderate reduction in the association of transcription factors with Pol III-transcribed loci (Fig. 6). It therefore appears that, while Iwr1 has a modest effect on transcription factor association, it exerts a more significant influence on the process of transcription itself. Accordingly, we observed a reduced rate of Pol III-transcribed RNA synthesis in the *iwr1* mutant strain (Fig. 3). Interestingly, the occupancy profile of the Pol III general transcription factors in the *iwr1Δ* strain (namely decreased polymerase occupancy accompanied by a slight increase in TFIIIC occupancy) is reminiscent of that observed in yeast strains subjected to conditions repressive for Pol III transcription [30].

The reduction in both TBP and polymerase association with Pol II-transcribed loci in the *iwr1* mutant is indicative of a defect in preinitiation complex formation and hence likely in the rate of transcriptional initiation. Indeed, after the work in this paper was completed, it was demonstrated that Iwr1 is directly involved in the nuclear import of Pol II [23]. However, we observe no significant consequence for steady-state mRNA levels. This suggests that, in contrast with the situation at Pol III-transcribed loci, there is a compensatory mechanism, presumably at the level of transcript stability either during or after completion of the RNA, to regulate mRNA levels. A similar discordance between Pol II occupancy and RNA levels has also been observed in yeast cells lacking either the Swi/Snf nucleosome remodeling complex [31] or the Asf1 histone chaperone [32], suggestive of a general compensation mechanism that is not specific to Iwr1. Similarly, although TBP occupancy is lower at the Pol I-transcribed rDNA locus in the *iwr1* strain, we saw no decrease in Pol I-transcribed ribosomal RNA levels, including levels of precursor RNA (Fig. 8). Taken together, these results indicate that, although Iwr1 influences transcription factor occupancy at promoters controlled by all three RNA polymerases, *iwr1* mutant strains are specifically defective in accumulating Pol III-transcribed RNAs, thereby explaining the Pol III-specific phenotypes.

Although the direct role of Iwr1 in nuclear import of Pol II [23] can explain the observed reduction in Pol II preinitiation complexes, the mechanism by which Iwr1 affects preinitiation complexes containing either Pol I or Pol III is unclear. Iwr1 does not affect nuclear import of Pol I or Pol III [23], indicating that Iwr1 affects transcription by Pol I and Pol III by a different mechanism(s). In this regard, in the plant *Aradopsis thaliana*, the Iwr1 homolog affects transcription by Pol IV and Pol V, which are involved in RNA-directed DNA methylation [33]. It is unlikely that the reduction in Pol III- and Pol I-containing preinitiation complexes is due to a defect in transcription by Pol II, because mRNA levels of all genes tested (including many encoding components of Pol III factors) are unaffected in an *iwr1* mutant strain.

Our efforts to perform ChIP on the Iwr1 protein itself with either N- or C-terminal epitope tags were not successful, suggesting that Iwr1 may not function directly at promoters or that its interaction is transient. The likelihood of an indirect function or transient interaction is supported by the low co-precipitation efficiency between Iwr1 and Pol III, and by the displacement of Iwr1 from Pol II by transcription initiation factors and DNA [23]. Interestingly, Iwr1 has been found in large-scale experiments to have genetic interactions with a significant number of chromatin modifying and remodeling proteins, including several components of the Swi/Snf complex, four members of the Swr1 complex, which exchanges histone variant H2AZ for H2A, and H2AZ itself [34]. A connection of Iwr1 to chromatin structure is further suggested by the compensatory mechanism for mRNA levels that is also observed in strains lacking Swi/Snf or Asf1 [31], [32]. These observations lead to the speculation that the effect of Iwr1 on TBP recruitment might be attributable to a possible influence on promoter accessibility mediated through these interactions with chromatin-associated proteins. Alternatively, Iwr1 might affect the nuclear import of TBP, the TBP-containing complex TFIIIB that is required for transcription by Pol III, or other factors affecting transcription and/or chromatin structure.

## Supporting Information

Methods S1 Additional methodological details beyond those in the Materials and Methods section.
(PDF)

## References

[1] Geiduschek EP, Kassavetis GA (2001). The RNA polymerase III transcription apparatus.. J Mol Biol.

[2] Hahn S (2004). Structure and mechanism of the RNA polymerase II transcription machinery.. Nat Struct Mol Biol.

[3] Russell J, Zomerdijk JC (2006). The RNA polymerase I transcription machinery.. Biochem Soc Symp.

[4] Schramm L, Hernandez N (2002). Recruitment of RNA polymerase III to its target promoters.. Genes Dev.

[5] Thomas MC, Chiang CM (2006). The general transcription machinery and general cofactors.. Crit Rev Biochem Mol Biol.

[6] Camier S, Gabrielsen O, Baker R, Sentenac A (1985). A split binding site for transcription factor tau on the tRNA3Glu gene.. Embo J.

[7] Struhl K (1999). Fundamentally different logic of gene expression in eukaryotes and prokaryotes.. Cell.

[8] Cormack BP, Struhl K (1992). The TATA-binding protein is required for transcription by all three nuclear RNA polymerases in yeast cells.. Cell.

[9] Goodrich JA, Tjian R (1994). TBP-TAF complexes: selectivity factors for eukaryotic transcription.. Curr Opin Cell Biol.

[10] Juvon-Gershon T, Hsu JY, Theisen JW, Kadonaga JT (2008). The RNA polymerase II core promoter- the gateway to transcription.. Curr Opin Cell Biol.

[11] Sims RJ, Belotserkovskaya R, Reinberg D (2004). Elongation by RNA polymerase II: the short and long of it.. Genes & Dev.

[12] Paule MR, White RJ (2000). Survey and summary: transcription by RNA polymerases I and III.. Nucl Acids Res.

[13] Willis IM, Moir RD (2007). Integration of nutritional and stress signaling pathways by Maf1.. Trends Biochem Sci.

[14] Bjork GR (1995). Genetic dissection of synthesis and function of modified nucleosides in bacterial transfer RNA.. Prog Nucleic Acid Res Mol Biol.

[15] Keith G, Glasser AL, Desgres J, Kuo KC, Gehrke CW (1990). Identification and structural characterization of O-beta-ribosyl-(1″----2′)-adenosine-5″-phosphate in yeast methionine initiator tRNA.. Nucleic Acids Res.

[16] Forster C, Chakraburtty K, Sprinzl M (1993). Discrimination between initiation and elongation of protein biosynthesis in yeast: identity assured by a nucleotide modification in the initiator tRNA.. Nucl Acids Res.

[17] Kiesewetter S, Ott G, Sprinzl M (1990). The role of modified purine 64 in initiator/elongator discrimination of tRNA(iMet) from yeast and wheat germ.. Nucleic Acids Res.

[18] Astrom SU, Bystrom AS (1994). Rit1, a tRNA backbone-modifying enzyme that mediates initiator and elongator tRNA discrimination.. Cell.

[19] Åström SU, Nordlund ME, Erickson FL, Hannig EM, Byström AS (1999). Genetic interactions between a null allele of the *RIT1* gene encoding an initiator tRNA-specific modification enzyme and genes encoding translation factors in *Saccharomyces cerevisiae*.. Mol Gen Genet.

[20] Gavin AC, Bosche M, Krause R, Grandi P, Marzioch M (2002). Functional organization of the yeast proteome by systematic analysis of protein complexes.. Nature.

[21] Krogan NJ, Cagney G, Yu H, Zhong G, Guo X (2006). Global landscape of protein complexes in the yeast Saccharomyces cerevisiae.. Nature.

[22] Peiro-Chova L, Estruch F (2009). The yeast RNA polymerase II-associated factor Iwr1 is involved in the basal and regulated transcription of specific genes.. J Biol Chem.

[23] Czeko E, Seizl M, Augsberger C, Mielke T, Cramer P (2011). Iwr1 directs RNA polymerase II nuclear import.. Mol Cell.

[24] Bender A, Pringle JR (1991). Use of a screen for synthetic lethal and multicopy suppressee mutants to identify two new genes involved in morphogenesis in Saccharomyces cerevisiae.. Mol Cell Biol.

[25] Kuras L, Struhl K (1999). Binding of TBP to promoters *in vivo* is stimulated by activators and requires Pol II holoenzyme.. Nature.

[26] Kranz JE, Holm C (1990). Cloning by function: an alternative approach for identifying yeast homologs of genes from other organisms.. Proc Natl Acad Sci USA.

[27] Carles C, Treich I, Bouet F, Riva M, Sentenac A (1991). Two additional common subunits, ABC10 alpha and ABC10 beta, are shared by yeast RNA polymerases.. J Biol Chem.

[28] Geiduschek EP, Kassavetis GA (2001). The RNA polymerase III transcription apparatus.. J Mol Biol.

[29] Åström SU, Byström AS (1994). Rit1, a tRNA backbone-modifying enzyme that mediates initiator and elongator tRNA discrimination.. Cell.

[30] Roberts DN, Stewart AJ, Huff JT, Cairns BR (2003). The RNA polymerase III transcriptome revealed by genome-wide localization and activity-occupancy relationships.. Proc Natl Acad Sci USA.

[31] Schwabish MA, Struhl K (2007). The Swi/Snf complex is important for histone eviction during transcriptional activation and RNA polymerase II elongation *in vivo*.. Mol Cell Biol.

[32] Schwabish MA, Struhl K (2006). Asf1 mediates histone eviction and deposition during elongation by RNA polymerase II.. Mol Cell.

[33] Kanno T, Bucher E, Daxinger L, Huettel B, Kreil DP (2010). RNA-directed DNA methylation and plant development require an IWR1-type transcription factor.. EMBO Rep.

[34] Collins SR, Miller KM, Maas NL, Roguev A, Fillingham J (2007). Functional dissection of protein complexes involved in yeast chromosome biology using a genetic interaction map.. Nature.

