# Supplementary material for: Iwr1 Protein Is Important for Preinitiation Complex Formation by All Three Nuclear RNA Polymerases in Saccharomyces cerevisiae
Source: PLoS One. 2011 Jun 10;6(6):e20829. doi: 10.1371/journal.pone.0020829 (PMC3112208; doi:10.1371/journal.pone.0020829)
Supplement: Methods S1 — Additional methodological details beyond those in the Materials and Methods section. (PDF) [file pone.0020829.s001.pdf]

SUPPLEMENTARY METHODS

Screen for mutants that require the *RIT1* gene for growth: The genetic screen utilized to identify mutants requiring *RIT1* for growth was based on a colony sectoring assay as described previously (BENDER and PRINGLE 1991; KRANZ and HOLM 1990). Candidate synthetic-lethal strains were crossed to UMY2395 and investigated for dominance/recessiveness and for 2:2 segregation of the non-sectoring phenotype. A YCp50 genomic library (ROSE *et al.* 1987) was used to transform *iwr1-2* (UMY2299), *rpc160-101* (UMY2304), and *rpb5-101* (UMY2309) mutants, and transformants that could lose the *RIT1* plasmid were identified. YCp50 plasmids isolated from transformants lacking *RIT1* were partially sequenced, and the DNA sequence was used for homology searches against the *S. cerevisiae* genome. To confirm that the mutations in UMY2299 and UMY2304 were genetically linked to the *IWR1* and *RPC160* loci, we integrated a *URA3* marker at the corresponding wild-type locus in *rit1*Δ strains, generating UMY2448 and UMY2332. These strains were crossed to each mutant (UMY2299 and UMY2304) and tetrad analysis showed co-segregation of the Ura^+ and sectoring phenotypes.

Strains, media, and genetic procedures: The source and genotypes of yeast strains used in this study are listed in table 1. The *E. coli* strain used was DH5α (Bethesda Research Laboratories). Yeast transformation (GIETZ *et al.* 1992), media, and genetic procedures have been described (BURKE *et al.* 2000). Strains deleted for the *RIT1* gene (UMY2395 and UMY2396) were constructed by the two-step gene replacement method (SCHERER and DAVIS 1979). Plasmid pRS306-*rit1::TRP1* (p1112) was linearized with *Sph*I and targeted to the *RIT1* locus (ORR-WEAVER *et al.* 1981) in strains UMY2219 and UMY2220. Transformation

of strain UMY2396 with p1119 generated strain UMY2418. The wild-type *IWR1* gene was replaced by mutant *iwr1* alleles in strain UMY2418 using the two-step gene replacement method. Plasmid pRS306-*iwr1-1* (p1331), pRS306-*iwr1-2* (p1332) and pRS306-*iwr1-3* (p1333) were linearized with *Bgl*II and targeted to the *IWR1* locus. The UMY2808 (*iwr1-2*) strain was obtained from a cross between UMY2451 and UMY2219. To obtain strain UMY2448 (*IWR1*::pRS306-*IWR1*), plasmid pRS306-*IWR1* (p1275) was digested with *Bgl*II and integrated at the *IWR1* locus in UMY2395. The original *rpc160-101 rit1::TRP1* p1119 mutant was backcrossed twice with UMY2395 generating UMY2469. An *rpc160-101* strain (UMY2802) was obtained from a cross between UMY2469 and UMY2219. Strain *RPC160*::pRS306-*RPC160* (UMY2332) was constructed by digesting plasmid pRS306-*RPC160* (p1187) with *Sph*I and integrating it at the *RPC160* locus in UMY2395. The *RPB5* gene was replaced with the *rpb5-101* allele from the original mutant in UMY2418 using the two-step gene replacement method (SCHERER and DAVIS 1979). To target the *rpb5-101* allele a *Mfe*I site was used to linearize the plasmid pRS306-*rpb5-101* (p1285). The *rpb5-101* (UMY2804) strain was obtained in a cross between *rpb5-101 rit1::TRP1* p1119 strain (UMY2426) and UMY2219. By crossing *rpc160-101* (UMY2802) or *rpb5-101* (UMY2804) with *iwr1-2* (UMY2808) carrying the pRS316-*IWR1* we constructed *rpc160-101 iwr1-2* (UMY2984) and *rpb5-101 iwr1-2* (UMY2986). The *maf1::KanMX4* strain (UMY2975) was constructed using a one step PCR mediated method (LONGTINE *et al.* 1998). An *iwr1-2 maf1::KanMX4* double mutant (UMY3059) was generated in a cross between strains UMY2975 and UMY2808. Strains, *IWR1-13MYC* (UMY3034), *RPC160-3HA* (UMY3031) and *IWR1-3HA* (UMY3032) were constructed using a one step PCR mediated method (LONGTINE *et al.* 1998). The *RPC160-3HA IWR1-13MYC* (UMY3035) strain was generated

in a cross between strains *RPC160-3HA* (UMY3031) and *IWR1-13MYC* (UMY3034). The *RPC160-3HA iwr1-2* (UMY3241) strain was generated in a cross between strains *RPC160-3HA* (UMY3031) and *iwr1-2* (UMY2808). Sequence analyses on chromosome IV revealed that *iwr1-1* has an insertion of an adenine at position 255085, the *iwr1-2* mutant allele carries an insertion of a thymidine at position 254821, and in the *iwr1-3* mutant there is a substitution from a guanine to a thymine at position 254368.

Plasmid constructions: DNA manipulations, plasmid preparations, and bacterial transformations were performed according to standard protocols. The plasmid pRS316-*IWR1* (p1251) was constructed by cloning a *Hin*dIII/*Xba*I *IWR1* fragment isolated from a library plasmid complementing *iwr1-2* mutant into the corresponding sites of pRS316. Plasmid pRS316-*RPC160* (p1615) was constructed by cloning an *Aha*II/*Xho*I fragment from an YCp50 library plasmid complementing the *rpc160-101* mutant, into the *Cla*I/*Xho*I sites of pRS316. For further information regarding plasmids used in this paper please contact the corresponding author.

Immunofluorescence: To localize Iwr1, cells were grown in 5 ml YEPD at 30° to an OD_{600} of 0.3, 670 μl formaldehyde (37%) was added and the cells were incubated for 40 min at RT. Cells were collected and washed once with 1 x PBS, pH 7.4. Immunofluorescence was done as described (BURKE *et al.* 2000). The primary antibody, mouse anti-HA (12CA5), was diluted 1:2000. The secondary antibody, goat anti-mouse linked to Cy3 (PA43002, Amersham Biosciences), was diluted 1:200. Cells were viewed in a Zeiss Axioskope 50 microscope using a 100x objective. Images were acquired using a Hamamatsu-digital camera (C4742-95).

Polysome profiles: Cells were grown in 200 ml at 30° in selective medium to an OD_{600} 0.4. Cycloheximide was added (100 µg/ml) 5 min before transferring the culture to an ice water bath for 15 min. Cells were collected at 4°, washed twice in ice-cold Breaking buffer (Bb; 20 mM Tris-HCl pH 7.4, 10 mM $MgCl_2$, 100 mM KCl, 0.5 mM DTT, 100 µg/ml cycloheximide). The cells were resuspended in 1 volume of Bb, followed by addition of 1 volume of glass beads, and cells were disrupted by 6 x 20 sec on a vortex mixer, and the insoluble material was pelleted by centrifugation at 10,000 x g for 5 min at 4°. The Supernatant was transferred to a microfuge tube and subjected to a second centrifugation at 10,000 x g for 20 min at 4°. The Supernatant was applied to a 12 ml linear 10 to 45 % sucrose gradient prepared in Bb lacking cycloheximide and centrifuged for 2.5 hrs at 40,000 rpm in a SW41 rotor at 4°. The gradients were collected from the top, and A_{254} absorbances were monitored with the ISCO detection system.

Northern blots: Cells were grown in 50 ml YEPD at 30° to an OD_{600} of 0.3 and total RNA prepared in the presence of glass beads. Northern blot analysis was performed as described (JOHANSSON and BYSTRÖM 2004). Oligonucleotides used for detection in Northern blots were, 5'-GGACATCAGGGTTATGAGCC-3'($tRNA_i^{Met}$), 5'-TGCTCCAGGGGAGGTTCGAAC-3' ($tRNA_m^{Met}$) and 5'-GCGTTGTTCATCGAT-3' (5.8S rRNA). Northern blots were visualized and quantified by phosphorimager analysis. Unless otherwise stated,10 µg total RNA was separated on 8 % polyacrylamide 8 M urea gel for Northern blotting. Gels were stained with ethidium bromide (10 µg/ml) for 10 min and destained in 1 x TBE for 10 min. RNA were visualized and quantified by using a BioRad Fluor-S™ MultiImager and the QuantityOne-4.2.3 software.

rtPCR: Total RNA was isolated from exponentially growing wild-type and *iwr1*Δ strains by the acid phenol method. The RNA was purified and treated with DNase I on Qiagen RNeasy columns, and first-strand cDNA synthesis was performed with random hexamers and Superscript III reverse transcriptase (Invitrogen) on 1 μg of total RNA. The relative representation of specific loci in this material was assayed by quantitative PCR in real-time on an Applied Biosystems 7500 machine.

***In vivo* labeling of RNA:** In labeling experiments, cells were grown to an OD_{600} of 0.8 in 120 ml SC-uracil medium at 30° before 125 μCi of ^{3}H-Uridine was added (33 Ci/mmol, Amersham Biosciences). Samples (20 ml) were collected after 0, 5, 10, 20, and 40 min. Total RNA was prepared and separated on an 8 % polyacrylamide 8 M urea gel. The gel was stained with ethidium bromide, quantified, soaked in NAMP100 Amplifyer for 15 min (Amersham Biosciences), dried onto 3 MM Whatman paper, and exposed to film. Signals were quantified using QuantityOne-4.2.3 software (BioRad), and the rate of synthesis for each RNA type was calculated. For the pulse-chase experiment, cells were grown to an OD_{600} of 0.8 in 50 ml SC-uracil medium at 30° and pulse-labeled using 125 μCi of ^{3}H-Uridine (33 Ci/mmol Amersham Biosciences) for 45 min. Cells were collected and resuspended in 250 ml pre-warmed SC medium containing excess (2 mM) uracil to begin the chase. Samples (40 ml) were collected after 0, 1, 2, 3, and 4 hrs. RNA was prepared, separated, and quantified as described above.

Immunoprecipitation: Cells were grown in 50 ml YEPD at 30° to an OD_{600} of 0.8, and total protein was prepared in buffer 1 (0.15 M Tris-HCl, pH 7.8, 50 mM KAc, 20 % glycerol, 1 mM EDTA, 1x Protease inhibitors, 0.5 mM DTT) by vortex mixing, 8 x 30 sec, in the presence of glass beads. Protein concentration was determined with BioRad protein-

assay kit. In immunoprecipitation experiments, 50 μg proteins was incubated for 2 hrs with agarose beads (Sepharose 4 Fast Flow, Amersham Biosciences) in a rotating chamber to reduce nonspecific binding. Beads were recovered and the supernatant transferred to tubes containing agarose beads linked to either anti-HA or anti-MYC antibody (Sigma A2095 or M5546). Samples were incubated 2 hrs at 4° in a rotating chamber. Beads were recovered and washed six times using 1 ml of buffer 2 (0.15 M Tris-HCl, pH 7.8, 50 mM KAc, 20 % glycerol, 1 mM EDTA, 1x Protease inhibitors, 0.5 mM DTT, 0.5 % Triton-X100), and proteins bound to the beads were recovered by incubating samples at 100° for 3 min in 1x loading buffer. Recovered tagged proteins were detected using standard western blot techniques.

Chromatin immunoprecipitation: Yeast strains BY4741 and the isogenic strain containing an *iwr1* null allele RN3812 were obtained from Invitrogen. Cells were cultured in YEPD medium to an OD_{600} of 0.6, crosslinked with 1 % formaldehyde, washed, and harvested as previously described (KURAS and STRUHL 1999). Total chromatin was isolated from these cells essentially as described (KURAS and STRUHL 1999) and sonicated to an average DNA fragment size between 300 and 500 bp. Chromatin immunoprecipitation was performed using antibodies against Tfc4, Rpc34 (both antibodies kindly supplied by Steve Hahn), Bdp1 (kindly provided by Ian Willis), and Rpb1 (8WG16 antibody from Covance). Immunoprecipitated DNA and total input control DNA were assayed by real-time quantitative PCR using the Applied Biosystems 7300 Real-time PCR System. Immunoprecipitation efficiency was determined for each locus by dividing the yield of PCR product in the immunoprecipitation sample by the amount of product obtained from the input control. Relative occupancy values were determined by dividing the immunoprecipitation

efficiency at each locus by the immunoprecipitation efficiency at a negative control locus (either the middle of the *POL1* ORF or an ORF-free region of chromosome V). The occupancy value of the negative control, 1.0, was subtracted from all values to yield a baseline of 0. All occupancy values were normalized to set the occupancy of each factor in the wild-type strain at *tC(GCA)B* locus equal to 100 units. All experiments were performed a minimum of three times.

LITERATURE CITED

BENDER, A., and J. R. PRINGLE, 1991 Use of a screen for synthetic lethal and multicopy suppressee mutants to identify two new genes involved in morphogenesis in *Saccharomyces cerevisiae*. Mol Cell Biol **11:** 1295-1305.

BURKE, D., D. DAWSON, T. STEARNS and COLD SPRING HARBOR LABORATORY, 2000 *Methods in yeast genetics : a Cold Spring Harbor Laboratory course manual*. Cold Spring Harbor Laboratory Press, Plainview, N.Y.

GIETZ, D., A. ST JEAN, R. A. WOODS and R. H. SCHIESTL, 1992 Improved method for high efficiency transformation of intact yeast cells. Nucleic Acids Res **20:** 1425.

JOHANSSON, M. J. O., and A. S. BYSTRÖM, 2005 Transfer RNA modifications and modifying enzymes in *Saccharomyces cerevisiae*, pp. 87-120 in *Fine-tuning of RNA functions by modification and editing*., edited by H. GROSJEAN. Springer-Verlag, New-York.

KRANZ, J. E., and C. HOLM, 1990 Cloning by function: an alternative approach for identifying yeast homologs of genes from other organisms. Proc Natl Acad Sci U S A **87:** 6629-6633.

KURAS, L., and K. STRUHL, 1999 Binding of TBP to promoters in vivo is stimulated by activators and requires Pol II holoenzyme. Nature **399:** 609-613.

LONGTINE, M. S., A. MCKENZIE, 3RD, D. J. DEMARINI, N. G. SHAH, A. WACH *et al.*, 1998 Additional modules for versatile and economical PCR-based gene deletion and modification in *Saccharomyces cerevisiae*. Yeast **14:** 953-961.

ORR-WEAVER, T. L., J. W. SZOSTAK and R. J. ROTHSTEIN, 1981 Yeast transformation: a model system for the study of recombination. Proc Natl Acad Sci U S A **78:** 6354-6358.

ROSE, M. D., P. NOVICK, J. H. THOMAS, D. BOTSTEIN and G. R. FINK, 1987 A *Saccharomyces cerevisiae* genomic plasmid bank based on a centromere-containing shuttle vector. Gene **60:** 237-243.

SCHERER, S., and R. W. DAVIS, 1979 Replacement of chromosome segments with altered DNA sequences constructed in vitro. Proc Natl Acad Sci U S A **76:** 4951-4955.
